# Queensland diabetic foot hospitalisations (2005-10): in what state is our foot hospital problem?

**DOI:** 10.1186/1757-1146-6-S1-P8

**Published:** 2013-05-31

**Authors:** Peter A Lazzarini, Sharon R O’Rourke, Anthony W Russell, Patrick H Derhy, Maarten C Kamp

**Affiliations:** 1 Allied Health Research Collaborative, Metro North Hospital & Health Service, Queensland Health, Brisbane, Queensland, 4032, Australia; 2 Department of Podiatry, Metro North Hospital & Health Service, Queensland Health, Brisbane, Queensland, 4032, Australia; 3 School of Clinical Sciences, Queensland University of Technology, Brisbane, Queensland, 4059, Australia; 4 Cairns Diabetes Centre, Queensland Health, Cairns, Queensland, 4870, Australia; 5 Department of Diabetes & Endocrinology, Princess Alexandra Hospital, Brisbane, Queensland, 4012, Australia; 6 Diamantina Institute, The University of Queensland, Brisbane, Queensland, 4072, Australia; 7 Centre for Healthcare Improvement, Queensland Health, Brisbane, Queensland, 4029, Australia; 8 School of Medicine, The University of Queensland, Brisbane, Queensland, 4072, Australia; 9 Department of Endocrinology, Metro North Hospital & Health Service, Queensland Health, Brisbane, Queensland, 4029, Australia

## Background

Diabetes foot complications are a leading cause of overall avoidable hospital admissions. Since 2006, the Queensland Diabetes Clinical Network has implemented programs aimed at reducing diabetes-related hospitalisation. The aim of this retrospective observational study was to determine the incidence of diabetes foot-related hospital admissions in Queensland from 2005 to 2010.

## Methods

Data on all primary diabetes foot-related admissions in Queensland from 2005-2010 was obtained using diabetes foot-related ICD-10-AM (hospital discharge) codes. Queensland diabetes foot-related admission incidences were calculated using general population data from the Australian Bureau of Statistics. Furthermore, diabetes foot-related sub-group admissions were analysed. Chi-squared tests were used to assess changes in admissions over time.

## Results

Overall, 24,917 diabetes foot-related admissions occurred, resulting in the use of 260,085 bed days or 1.4% of all available Queensland hospital bed days (18,352,152). The primary reasons for these admissions were foot ulcers (49.8%), cellulitis (20.7%), peripheral vascular disease (17.8%) and osteomyelitis (3.8%). The diabetes foot-related admission incidence among the general population (per 100,000) reduced by 22% (103.0 in 2005, to 80.7 in 2010, *p* < 0.001); bed days decreased by 18% (1,099 to 904, *p* < 0.001).

## Conclusion

Diabetes foot complications appear to be the primary reason for 1.4 out of every 100 hospital beds used in Queensland. There has been a significant reduction in the incidence of diabetes foot-related admissions in Queensland between 2005 and 2010. This decrease has coincided with a corresponding decrease in amputations and the implementation of several diabetes foot clinical programs throughout Queensland.

